# VSTM-v1, a potential myeloid differentiation antigen that is downregulated in bone marrow cells from myeloid leukemia patients

**DOI:** 10.1186/s13045-015-0118-4

**Published:** 2015-03-15

**Authors:** Min Xie, Ting Li, Ning Li, Jinlan Li, Qiumei Yao, Wenling Han, Guorui Ruan

**Affiliations:** Peking University People’s Hospital and Institute of Hematology, 11 Xi-Zhi-Men South Street, Beijing, 100044 China; Peking University Center for Human Disease Genomics, Department of Immunology, Key Laboratory of Medical Immunology, Ministry of Health, School of Basic Medical Sciences, Peking University Health Science Center, 38 Xueyuan Road, Beijing, 100191 China

**Keywords:** Acute myeloid leukemia, Leukocyte differentiation antigen, VSTM1, Methylation, Biomarker

## Abstract

Leukocyte differentiation antigens often represent important markers for the diagnosis, classification, prognosis, and therapeutic targeting of myeloid leukemia. Herein, we report a potential leukocyte differentiation antigen gene *VSTM1* (*V-set and transmembrane domain-containing 1*) that was downregulated in bone marrow cells from leukemia patients and exhibited a higher degree of promoter methylation. The expression level of its predominant encoded product, VSTM1-v1, was positively correlated with myeloid cell maturation state. Restoration of VSTM1-v1 expression inhibited myeloid leukemia cells’ growth. Therefore, VSTM1-v1 might represent an important myeloid leukocyte differentiation antigen and provide a potential target for the diagnosis and treatment of leukemia.

**Electronic supplementary material:**

The online version of this article (doi:10.1186/s13045-015-0118-4) contains supplementary material, which is available to authorized users.

## To the Editor

Acute myeloid leukemia (AML) and chronic myeloid leukemia (CML) are myeloid blood cell malignancies that show great heterogeneity. *VSTM1* (*V-set and transmembrane domain-containing 1*) encodes a potential leukocyte differentiation antigen that is highly expressed in myeloid cells, but silenced in multiple leukemia cell lines [1]. To determine whether it plays a role in leukemogenesis, we characterized its expression pattern and function in bone marrow cells from AML/CML patients and myeloid leukemia cell lines.

We measured *VSTM1* expression in leukemia cell lines and bone marrow biopsies from leukemia patients using qRT-PCR. *VSTM1* was downregulated or silenced in all cell lines tested (Additional file 1: Table S1). Compared to healthy donors (HDs), *VSTM1* was downregulated in AML (Table 1). Additionally, in CML-AP/BC (accelerated phase/blast crisis), which clinically behaves like AML, *VSTM1* expression levels were much lower than those in CML-CP (chronic phase, *P* = 0.003, Table 1). Analogous protein expression differences were found by Western blotting. Compared to HD bone marrow, VSTM1 was similarly expressed in CML-CP patients, whereas it was barely detectable in untreated AML patients. However, in AML patients who achieved complete remission, VSTM1 expression was completely restored (Additional file 2: Figure S1). Higher levels of *VSTM1* promoter methylation in bone marrow cells from AML patients compared to those from HDs were observed, which might contribute to its downregulation (Additional file 3: Figure S2).

**Table 1:** **The expression level of** ***VSTM1*** **in bone marrow cells from leukemia patients and healthy donors**

**Groups**	**MIC**	**Sample size**	**Mean ratio,** ***VSTM1*** **/** ***ABL***	***P*** **value** ^**a**^
HD		36	17.358 ± 17.904	
Untreated AML		145	4.333 ± 7.895	<0.001
	M1	3	0.374 ± 0.619	n.a.
	M2	72	5.292 ± 10.171	<0.001
	M3	29	2.884 ± 3.960	<0.001
	M4	24	4.814 ± 5.293	0.001
	M5	14	3.052 ± 4.867	<0.001
	M6	3	1.417 ± 2.205	n.a.
Untreated ALL		40	0.381 ± 0.755	<0.001
Untreated CML		57	5.479 ± 8.266	<0.001
	CP	38	7.743 ± 9.312	0.001
	AP/BC	19	0.950 ± 1.367	<0.001

*MIC* morphological, immunological, and cytogenetic classifications, *n.a.* not available due to a small sample size.

^a^The *P* value was calculated using Wilcoxon signed ranks test as compared to the HD group.

VSTM1-v1 is the most abundantly expressed gene product encoded by *VSTM1* [2] and is an ITIM-bearing immune receptor that negatively regulates neutrophil activity [3-5]. We used flow cytometry to divide bone marrow cells into subpopulations based on CD45 levels and SSC [6], and found that VSTM1-v1 was highly expressed in mature granulocytes and monocytes from HDs (94.01 ± 6.80% positive with a mean fluorescence intensity (MFI) of 167.61 ± 90.95), but was much less abundant in naive cells (37.14 ± 14.47% with a MFI of 46.70 ± 23.51, *n* = 27; *P* <0.001). Moreover, the percentage of VSTM1-v1-positive cells among naive cells from AML patients (19.60 ± 21.09%, *n* = 52, including 4 M0/M1, 22 M2, 4 M3, 14 M4, and 8 M5) was even lower than that in HDs (*P* = 0.047). Therefore, we speculated that VSTM1-v1 expression might be associated with the maturity of myeloid cells. A combination of CD34/CD117/CD13/CD16 staining showed that VSTM1-v1 expression in myeloid cells was positively correlated with cell maturation state. Differences between any two continuous stages were significant (*n* = 11; *P* < 0.001; Table 2 and Additional file 4: Figure S3). Using CD16 and CD14 as phenotypic markers for mature granulocytes and monocytes, respectively, we found a similar result (Additional file 1: Table S2). This correlation was subsequently confirmed by increased *VSTM1* expression in bone marrow cells from APL patients and in NB4 cells after ATRA treatment *in vitro* (Additional file 5: Figure S4). These findings provide a potential reason why *VSTM1* expression levels were reduced so markedly in AML and CML-AP/BC patients.

**Table 2:** **The expression of VSTM1-v1 at various stages of myelocytic differentiation**

	**Myeloblasts**	**Promyelocytes**	**Myelocytes**	**Metamyelocytes**
CD markers	CD34^+^	CD34^−^, CD117^+^, CD16^−^	CD34^−^, CD117^−^, CD16^−^, CD13^+^	CD16^+^
VSTM1-v1-positive cells (%)	5.69 ± 2.69	11.48 ± 4.58	35.02 ± 11.58	61.63 ± 8.53
*P* value^a^	<0.001	<0.001	<0.001	

^a^The *P* value was calculated using a Wilcoxon signed ranks test as compared with the next stage.

Similar to its function in Jurkat cells [1], restoration of VSTM1-v1 expression in the myeloid leukemia cell lines K562 and MEG-01 also inhibited cell growth (Additional file 6: Figure S5). Moreover, when searching for clinical features that could be related to *VSTM1* expression in AML patients, we detected a higher expression level of *VSTM1* in *AML1-ETO*-positive patients (Additional file 1: Table S3 and Additional file 7: Figure S6). This chimeric oncogene in AML is often associated with a relatively favorable prognosis [7,8]. Considering the inhibitory effect of VSTM1-v1 on leukemia cell growth, we can hypothesize that VSTM1-v1 might enhance the cytotoxic effects of chemotherapeutics in patients with this genetic abnormality.

In conclusion, our findings suggest that VSTM1-v1 might be an important myeloid leukocyte differentiation antigen. Our elucidation of its expression pattern throughout myeloid cell differentiation and its effect on leukemia cell growth could help to establish it as a novel target for the development of diagnostics and treatments for myeloid leukemia.

## Additional files

Additional file 1: Table S1. The expression level of *VSTM1* in leukemia cell lines. **Table S2.** Correlations between VSTM1-v1 expression and phenotypic markers of mature granulocytes and monocytes. **Table S3.** Correlations between *VSTM1* expression and clinical features of AML patients. Additional file 2: Figure S1. Western blot analysis of VSTM1 expression in bone marrow cells. Bone marrow samples were from two healthy donors (N1 and N2), four AML patients who achieved complete remission (L1 and L2, by bone marrow transplantation; L3 and L4, by chemotherapy), four untreated AML patients (L5–8), and two untreated CML-CP patients (L9 and L10). Additional file 3: Figure S2. The *VSTM1* promoter was hypermethylated in AML patients. (A) Methylation-specific PCR (MSP) of the *VSTM1* promoter in bone marrow samples from eight AML patients (lanes 1–8). Among them, seven samples (lanes 1–3, and 5–8) were partially methylated and one (lane 4) was completely methylated; M, methylated; U, unmethylated. (B) Bisulfite genomic sequencing (BGS) of the *VSTM1* promoter in bone marrow samples from four healthy donors and four AML patients were carried out, which demonstrated that *VSTM1* underwent increased promoter methylation in AML patients (57/208, 27.40%) compared to healthy donors (18/208, 8.65%; *P* < 0.05). Representative results from a healthy donor (HD) and an AML patient are shown. Circles, CpG sites that were analyzed; rows of circles, an individual promoter allele that was cloned, randomly selected, and sequenced; filled circle, a methylated CpG site; open circle, an unmethylated CpG site. Additional file 4: Figure S3. Flow cytometric analysis of VSTM1-v1 expression in subpopulations of bone marrow cells from healthy donors. A representative result is shown. (A) CD45 intensity and side scatter (SSC) were used to set gates for lymphocytes (CD45^high^ SSC^low^), mature granulocytes (CD45^int^ SSC^high^), monocytes (CD45^high^ SSC^int^) and naive cells (CD45^int^ SSC^low^). The percentages of VSTM1-v1-positive cells in each population were determined. (B) The naive cell population in (A) was then further gated based on combinations of CD34, CD117, CD13, and CD16 staining and were classified into myeloblasts (CD34^+^), promyelocytes (CD34^−^CD117^+^CD16^−^), myelocytes (CD34^−^CD117^−^CD16^−^CD13^+^) and metamyelocytes (CD16^+^). The percentages of VSTM1-v1-positive cells in each population were determined. Additional file 5: Figure S4. VSTM1-v1 expression was restored in APL cells by ATRA treatment. (A) qRT-PCR for *VSTM1* expression in bone marrow cells from two APL patients that were untreated or treated with ATRA for 7 days *in vitro*. (B) Flow cytometric analysis of the percentage of VSTM1-v1-positive NB4 cells that were untreated or treated with ATRA for 5 days. CD11b expression was measured as a marker of differentiation. A representative result of three independent experiments is shown. (C) The correlation of the MFI of VSTM1-v1 and CD11b at various time points following treatments with different concentrations of ATRA was evaluated by Pearson’s correlation analysis (n = 17; r = 0.866; *P* < 0.001). Additional file 6: Figure S5. Overexpression of VSTM1-v1 inhibited K562 and MEG-01 cell growth. (A) Overexpressed VSTM1-v1 was detected on the cell surface by flow cytometry. Cell growth curves of K562 (B) and MEG-01 (C) cells were generated based on viable cell counting assays after VSTM1-v1 transfection. Error bars represent SD; *, *P* < 0.05, compared to the vector control at each time point. Representative results of at least three independent experiments are shown. Additional file 7: Figure S6. Correlations between *VSTM1* expression in AML patients and gender or the *AML1-ETO* fusion gene. (A) The expression level of *VSTM1* was higher in male than in female patients (median, 2.45 [0.00–171.90] vs. 1.03 [0.00–19.03]; *P* = 0.010). (B) The expression level of *VSTM1* was higher in *AML1-ETO*-positive (n = 46) than in *AML1-ETO*-negative (n = 44) patients (median, 2.58 [0.00–44.62] vs. 0.74 [0.00–54.08]; *P* < 0.001).

## Abbreviations

AML: Acute myeloid leukemia
CML: Chronic myeloid leukemia
CP: Chronic phase
AP: Accelerated phase
BC: Blast crisis
ALL: Acute lymphocytic leukemia
APL: Acute promyelocytic leukemia
ATRA: All-trans retinoic acid
WBC: White blood cell
Hb: Hemoglobin
Plt: Blood platelet count
