# Supplementary material for: VSTM-v1, a potential myeloid differentiation antigen that is downregulated in bone marrow cells from myeloid leukemia patients
Source: J Hematol Oncol. 2015 Mar 15;8:25. doi: 10.1186/s13045-015-0118-4 (PMC4405901; doi:10.1186/s13045-015-0118-4)
Supplement: Additional file 1: Table S1. — The expression level of VSTM1 in leukemia cell lines. Table S2. Correlations between VSTM1-v1 expression and phenotypic markers of mature granulocytes and monocytes. Table S3. Correlations between VSTM1 expression and clinical features of AML patients. [file 13045_2015_118_MOESM1_ESM.zip › 13045_2015_118_MOESM1_ESM.rtf]

VSTM-v1, a potential myeloid differentiation antigen that is downregulated in bone marrow cells from myeloid leukemia patients

Materials and methods

Patient samples
Bone marrow cells from 242 leukemia patients diagnosed at the Institute of Hematology, Peking University People's Hospital (Beijing, China) between Dec 2009 and Jun 2010 were collected and tested for VSTM1 expression. Diagnosis was established based on the widely used WHO criteria. In this cohort, there were 145 untreated AML cases, including 3 M1, 72 M2, 29 M3, 24 M4, 14 M5, and 3 M6 cases according to the FAB classification. There were also 29 cases of untreated ALL, 38 cases of untreated CML in a chronic phase (CML-CP), and 19 cases of CML in an accelerated phase/blast crisis (CML-AP/BC). Bone marrow cells from 36 healthy donors and 4 AML patients who were in complete remission were used as controls. Mononuclear cells were isolated from bone marrow cells using standard Ficoll–Hypaque density gradient centrifugation. All participants provided informed consent according to the Helsinki Declaration, and the protocol for this study was approved by the Ethics Committees of Peking University People's Hospital (Beijing, China).

Cell lines
The myeloid leukemia cell lines KG1, HL-60, NB4, THP-1, K562, MEG-01, and CEM (kindly provided by the Cell Bank Shanghai Institute of Cell Biology, Shanghai, China) were cultured in RPMI1640 medium (Life Technologies, Carlsbad, CA, USA) containing 10% fetal bovine serum (FBS, Gibco/Life Technologies), L-glutamine (4 mmol/L), penicillin (100 U/mL), and streptomycin (100 ìg/mL). Cells were grown at 37°C in a humidified 5% (v/v) CO2 atmosphere and were used for assays during the exponential growth phase.

All-trans retinoic acid (ATRA) treatment
Mononuclear cells isolated from the bone marrow of two AML-M3 (acute promyelocytic leukemia, APL) patients were maintained in vitro in RPMI1640 medium containing 10% FBS, 4.5 g/L glucose, 4 mmol/L L-glutamine, 100 U/mL penicillin, and 100 ìg/mL streptomycin; these cells were treated with ATRA (2 ìmol/L) for 7 days. The APL cell line NB4 was treated with ATRA (0.5, 1.0, 2.0, and 4.0 ìmol/L) for 1-5 days.

Reverse transcription-PCR (RT-PCR) and real-time quantitative PCR (qRT-PCR)
Total RNA was extracted from cells using TRIzol reagent (Life Technologies). Reverse transcription was performed according to standard protocols using a Reverse Transcription System (Promega, Madison, WI, USA). Semiquantitative PCR was performed as previously described [1]. GAPDH was amplified as an internal standard. Primers and the probe for qRT-PCR of VSTM1 were designed using Primer Express 2.0 software (Applied Biosystems, Foster City, CA, USA) as follows: VSTM1-FP, 5'-GCCGAGGCAGATTTATCCAA-3'; VSTM1-RP, 5'-CCTGGGTGGTGTCTGAAGCT-3'; and VSTM1-Probe, 5'-(FAM)CTCGACGGCAGACCCCCAAGG(BHQ)-3'. Primers and probes for AML1-ETO, PML-RARá, PRAME, WT1, and ABL1 were synthesized as previously reported [2],[3]. We performed qRT-PCR using an ABI PRISM® 7500 Sequence Detection System as previously described [4]. PCR mixtures contained 1× TaqMan® Universal PCR Master mix, 400 nM primers, 200 nM fluorescent probes, and 1 ìL cDNA. The copy numbers of ABL1 and VSTM1 were calculated using Ct values and standard curves that were generated. If the ABL1 copy number was ≥3×10^4^, samples were considered for quantitative detection. Expression of VSTM1 was normalized to the level of ABL1 and expressed as a relative value. All samples were examined in three independent experiments that were performed in triplicate.

DNA bisulfite treatment and promoter methylation analysis
Genomic DNA samples were extracted from cells using DNAzol (GenStar BioSolutions, Beijing, China) according to the manufacturer's instructions. Primers for detecting methylated or unmethylated alleles of the VSTM1 promoter and for bisulfite genomic sequencing (BGS) were designed and bisulfite modifications of DNA, methylation-specific PCR (MSP), and BGS were carried out as previously described [5]-[7]. 

Flow cytometry
Fluorescein-labeled anti-CD molecule antibodies were purchased from BD Bioscience (San Jose, CA, USA) and were used according to the manufacturer's instructions. FITC-conjugated rabbit anti-VSTM1 was kindly provided by Prof. Wenling Han (Peking University Health Science Center) and 2 ìg was used for each sample in 100 ìL FACS buffer (PBS containing 2% FBS) [8]. Bone marrow cells were stained with the combinations of either VSTM1-FITC, CD117-PE, CD45-PerCP, CD13-APC, CD34-PE-Cy7 and CD16-APC-Cy7 or VSTM1-FITC, CD117-PE, CD45-PerCP, CD33-APC, CD34-PE-Cy7 and CD14-APC-Cy7; stained cells were analyzed on a FACSort (Becton Dickinson, San Jose, CA, USA). Data were analyzed using FCS Express V3 software.

Protein extraction and western blotting
Cells were lysed in 20 mmol/L Tris–HCl (pH 7.5), 150 mmol/L NaCl, 1 mmol/L EDTA, 1% Triton X-100, and 1% protease inhibitor cocktail. Protein concentrations were determined using BCA protein assays (Pierce, Rockford, IL, USA). Whole cell lysates were then fractionated using 10% SDS–PAGE and electrotransferred onto polyvinylidene difluoride membranes (Hybond; GE Healthcare, Buckinghamshire, United Kingdom). Western blotting was carried out as previously described [5]. GAPDH was used as a lysate loading control.

Cell proliferation assay
K562 and MEG-01 cells were transfected with pcDNA3.1/Myc-His(-)B-VSTM1-v1 (termed pcDB-VSTM1-v1) or pcDNA3.1/Myc-His(-)B (termed pcDB) vector plasmids using Lipofectamine 2000 (Invitrogen) following the manufacturer's instructions. Six hours after transfection, the medium was refreshed and cells were plated in 96-well plates at a density of 5000 cells per well. Cell proliferation was analyzed by viable cell counting at the indicated time points. Results were obtained from at least three independent experiments in triplicate.

Statistical analysis
All analyses were performed using SPSS software 17.0 (Chicago, IL, USA). VSTM1 expression was correlated with clinical characteristics using general linear correlation analysis (for classification variables, such as gender) or the Spearman's rank correlation coefficient test for two variables (for continuous variables, such as age, white blood cell count [WBC] from peripheral blood, hemoglobin [Hb], blood platelet count [Plt], and genetic markers of leukemia [WT1, AML1-ETO, PML/RARα, PRAME, and CBFb/MYH11]). Differences between groups were evaluated using the Wilcoxon signed ranks test (Mann–Whitney rank sum test). A P-value<0.05 was considered to represent a statistically significant difference.


References

1. Guo X, Zhang Y, Wang P, Li T, Fu W, Mo X, Shi T, Zhang Z, Chen Y, Ma D, Han W: VSTM1-v2, a novel soluble glycoprotein, promotes the differentiation and activation of Th17 cells. Cell Immunol 2012, 278: 136-142. 
2. Beillard E, Pallisgaard N, van der Velden VH, Bi W, Dee R, van der Schoot E, Delabesse E, Macintyre E, Gottardi E, Saglio G, Watzinger F, Lion T, van Dongen JJ, Hokland P, Gabert J: Evaluation of candidate control genes for diagnosis and residual disease detection in leukemic patients using 'real-time' quantitative reverse-transcriptase polymerase chain reaction (RQ-PCR) - a Europe against cancer program. Leukemia 2003, 17: 2474-2486.
3. Qin YZ, Liu YR, Zhu HH, Li JL, Ruan GR, Zhang Y, Jiang Q, Jiang H, Li LD, Chang Y, Huang XJ, Chen SS: Different kinetic patterns of BCR-ABL1 transcript levels in imatinib-treated chronic myeloid leukemia patients after achieving complete cytogenetic response. Int J Lab Hematol 2008, 30: 317-323. 
4. Niu J, Li H, Zhang Y, Li J, Xie M, Li L, Qin X, Qin Y, Guo X, Jiang Q, Liu Y, Chen S, Huang X, Han W, Ruan G: Aberrant expression of CKLF-like MARVEL transmembrane member 5 (CMTM5) by promoter methylation in myeloid leukemia. Leuk Res 2011, 35: 771-776.
5. Li T, Guo XH, Wang WY, Mo XN, Wang PZ, Han WL: V‑set and transmembrane domain‑containing 1 is silenced in human hematopoietic malignancy cell lines with promoter methylation and has inhibitory effects on cell growth. Mol Med Rep 2015, 11:1344-1351.
6. Shao L, Cui Y, Li H, Liu Y, Zhao H, Wang Y, Zhang Y, Ng KM, Han W, Ma D, Tao Q: CMTM5 exhibits tumor suppressor activities and is frequently silenced by methylation in carcinoma cell lines. Clin Cancer Res 2007, 13: 5756-5762.
7. Wang Y, Li J, Cui Y, Li T, Ng KM, Geng H, Li H, Shu XS, Li H, Liu W, Luo B, Zhang Q, Mok TS, Zheng W, Qiu X, Srivastava G, Yu J, Sung JJ, Chan AT, Ma D, Tao Q, Han W: CMTM3, located at the critical tumor suppressor locus 16q22.1, is silenced by CpG methylation in carcinomas and inhibits tumor cell growth through inducing apoptosis. Cancer Res 2009, 69: 5194-5201.
8. Li T, Guo XH, Wang PZ, Song QS, Ma DL, Han WL: Preparation, Purification, and Characterization of the Polyclonal Antibody against Human VSTM1. Xi Bao Yu Fen Zi Mian Yi Xue Za Zhi 2012, 28: 1291-1294.
Table S1. The expression level of VSTM1 in leukemia cell lines.
Cell lines	MICa 	Repeat number	Mean ratio VSTM1: ABL1	
KG1	M1	4	0.4557±0.0959	
HL-60	M3	3	0	
NB4	M3	3	0.0011±0.0009	
THP-1	M5	4	0.0010±0.0012	
K562	CML-BC	3	0	
MEG-01	CML-BC	3	0	
CEM	T-ALL	3	0	
a MIC denotes the morphological, immunological and cytogenetic classification.

Table S2. Correlations between VSTM1-v1 expression and phenotypic markers of mature granulocytes and monocytes.
	Granulocytes	Monocytes	
	CD16dim	CD16+	CD16++	CD14-	CD14+	
VSTM1-v1-positive cells (%)	50.18±20.63	74.51±15.36	92.47±10.96	34.35±11.44	68.66±18.07	
P-valuea	0.005	0.005		0.004		
a The P-value is measured using Wilcoxon signed ranks test for comparisons with the next adjacent cell population.

Table S3. Correlations between VSTM1 expression and clinical features of AML patients.
Clinical factors	Sample size	Correlation coefficient	P -value	
Gender	145a	0.184	0.010b	
Age	145	0.040	0.649	
WBC	105	–0.007	0.947	
Hb	104	0.200	0.042b	
Plt	105	–0.043	0.661	
WT1	69	–0.116	0.344	
PRAME	31	–0.043	0.819	
AML1-ETO	90	0.361	< 0.001b	
PML-RARa	35	0.106	0.543	
CBFb-MYH11	11	0.164	0.631	
a 75 : 70 male to female
b The underlined P-values were considered to represent a statistically significant difference.
