# Supplementary figures and images for: VSTM-v1, a potential myeloid differentiation antigen that is downregulated in bone marrow cells from myeloid leukemia patients
Source: J Hematol Oncol. 2015 Mar 15;8:25. doi: 10.1186/s13045-015-0118-4 (PMC4405901; doi:10.1186/s13045-015-0118-4)

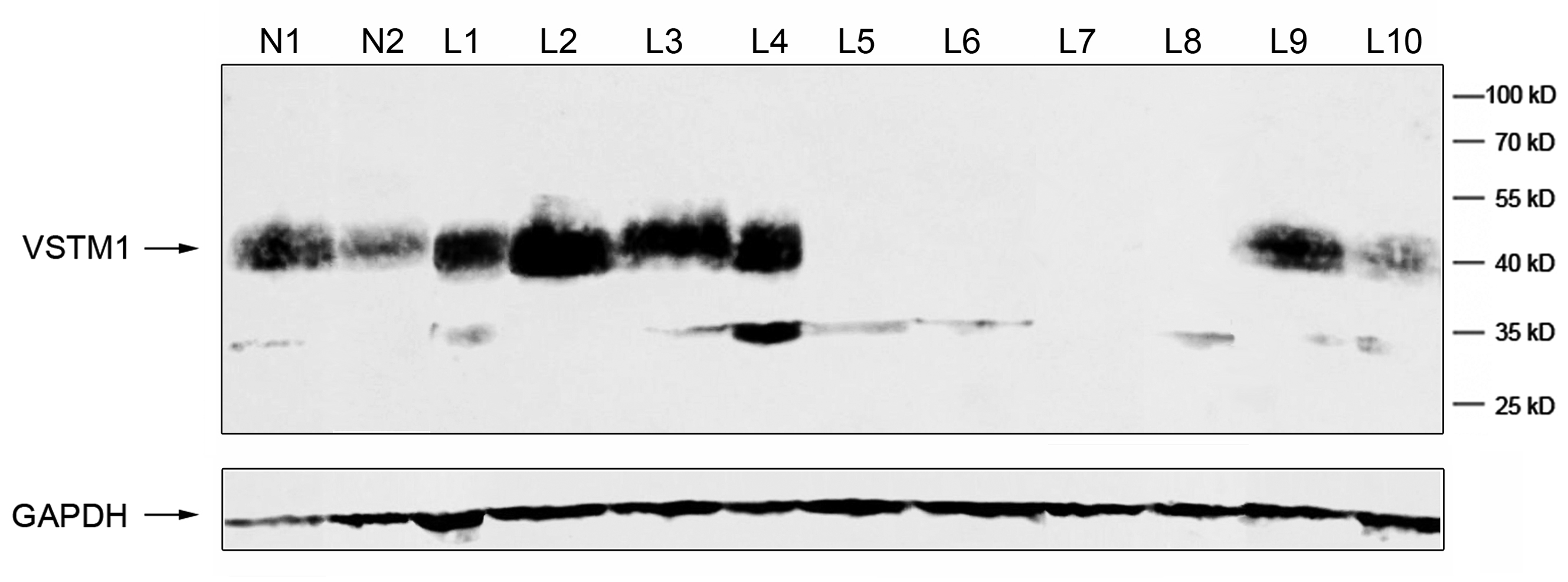

Supplement: Additional file 2: Figure S1. — Western blot analysis of VSTM1 expression in bone marrow cells. Bone marrow samples were from two healthy donors (N1 and N2), four AML patients who achieved complete remission (L1 and L2, by bone marrow transplantation; L3 and L4, by chemotherapy), four untreated AML patients (L5–8), and two untreated CML-CP patients (L9 and L10). [file 13045_2015_118_MOESM2_ESM.jpg]

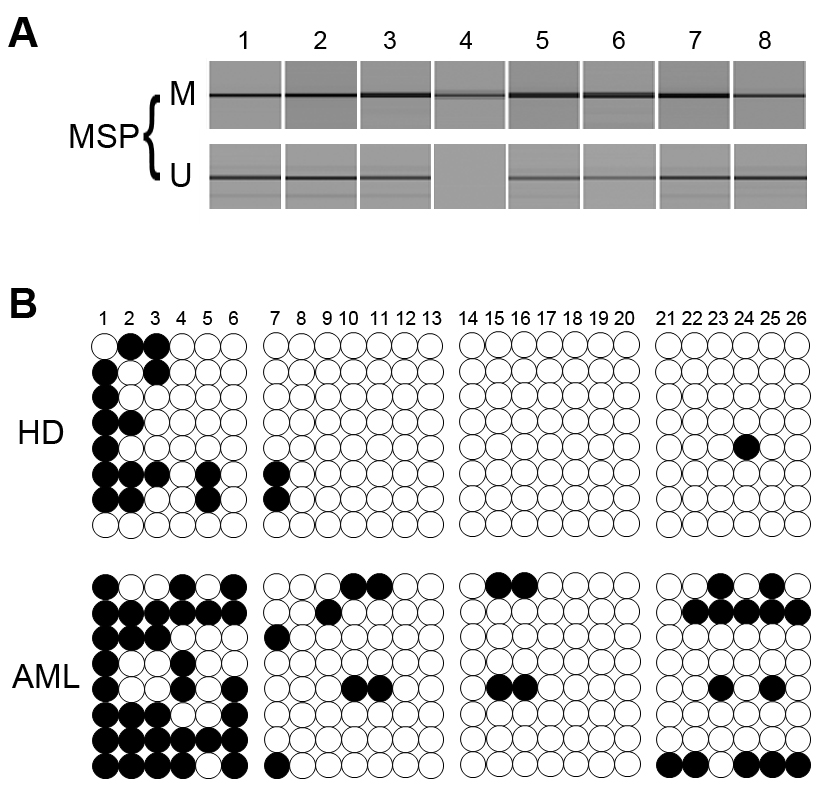

Supplement: Additional file 3: Figure S2. — The VSTM1 promoter was hypermethylated in AML patients. (A) Methylation-specific PCR (MSP) of the VSTM1 promoter in bone marrow samples from eight AML patients (lanes 1–8). Among them, seven samples (lanes 1–3, and 5–8) were partially methylated and one (lane 4) was completely methylated; M, methylated; U, unmethylated. (B) Bisulfite genomic sequencing (BGS) of the VSTM1 promoter in bone marrow samples from four healthy donors and four AML patients were carried out, which demonstrated that VSTM1 underwent increased promoter methylation in AML patients (57/208, 27.40%) compared to healthy donors (18/208, 8.65%; P < 0.05). Representative results from a healthy donor (HD) and an AML patient are shown. Circles, CpG sites that were analyzed; rows of circles, an individual promoter allele that was cloned, randomly selected, and sequenced; filled circle, a methylated CpG site; open circle, an unmethylated CpG site. [file 13045_2015_118_MOESM3_ESM.jpg]

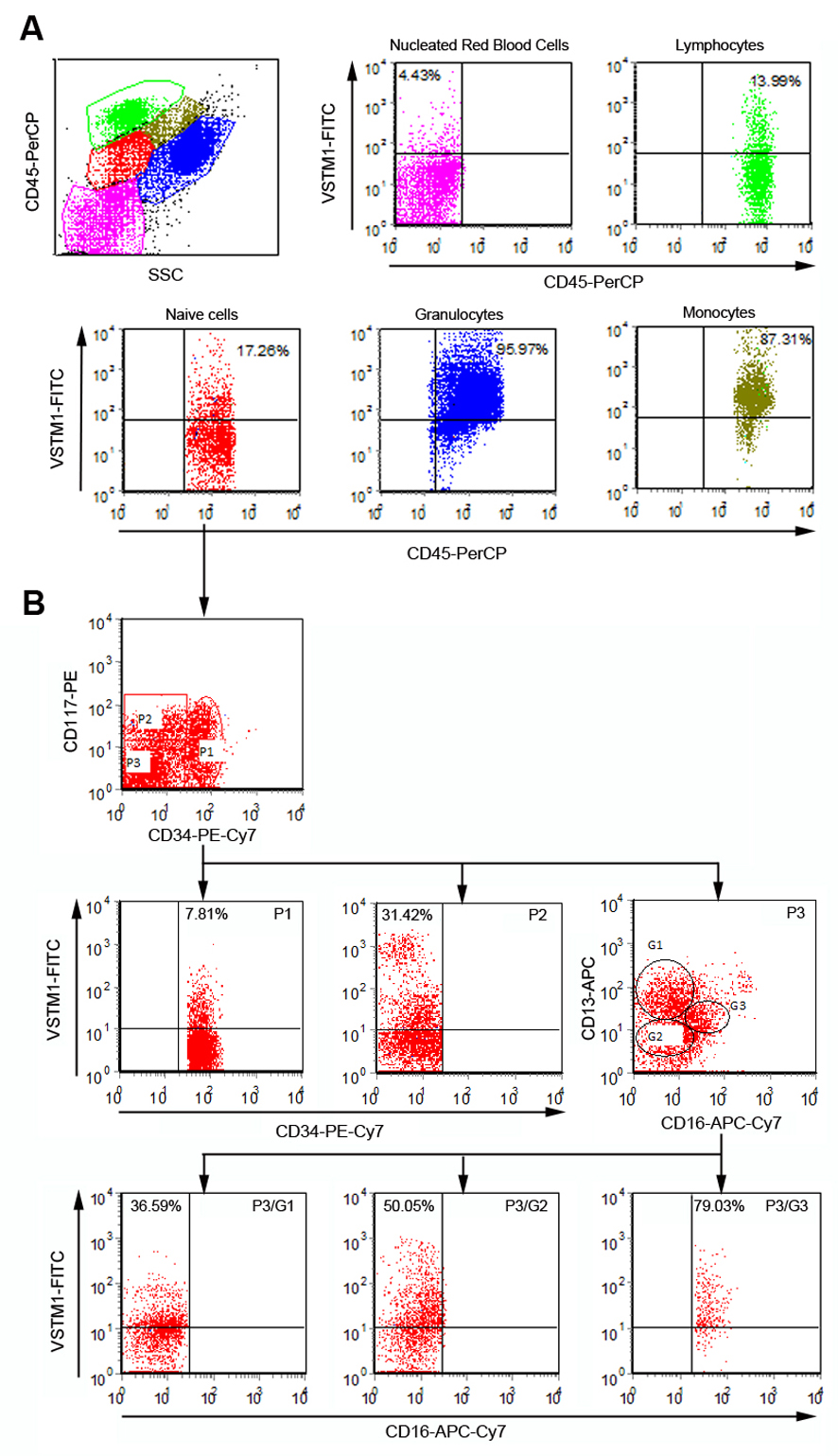

Supplement: Additional file 4: Figure S3. — Flow cytometric analysis of VSTM1-v1 expression in subpopulations of bone marrow cells from healthy donors. A representative result is shown. (A) CD45 intensity and side scatter (SSC) were used to set gates for lymphocytes (CD45high SSClow), mature granulocytes (CD45int SSChigh), monocytes (CD45high SSCint) and naive cells (CD45int SSClow). The percentages of VSTM1-v1-positive cells in each population were determined. (B) The naive cell population in (A) was then further gated based on combinations of CD34, CD117, CD13, and CD16 staining and were classified into myeloblasts (CD34+), promyelocytes (CD34−CD117+CD16−), myelocytes (CD34−CD117−CD16−CD13+) and metamyelocytes (CD16+). The percentages of VSTM1-v1-positive cells in each population were determined. [file 13045_2015_118_MOESM4_ESM.jpg]

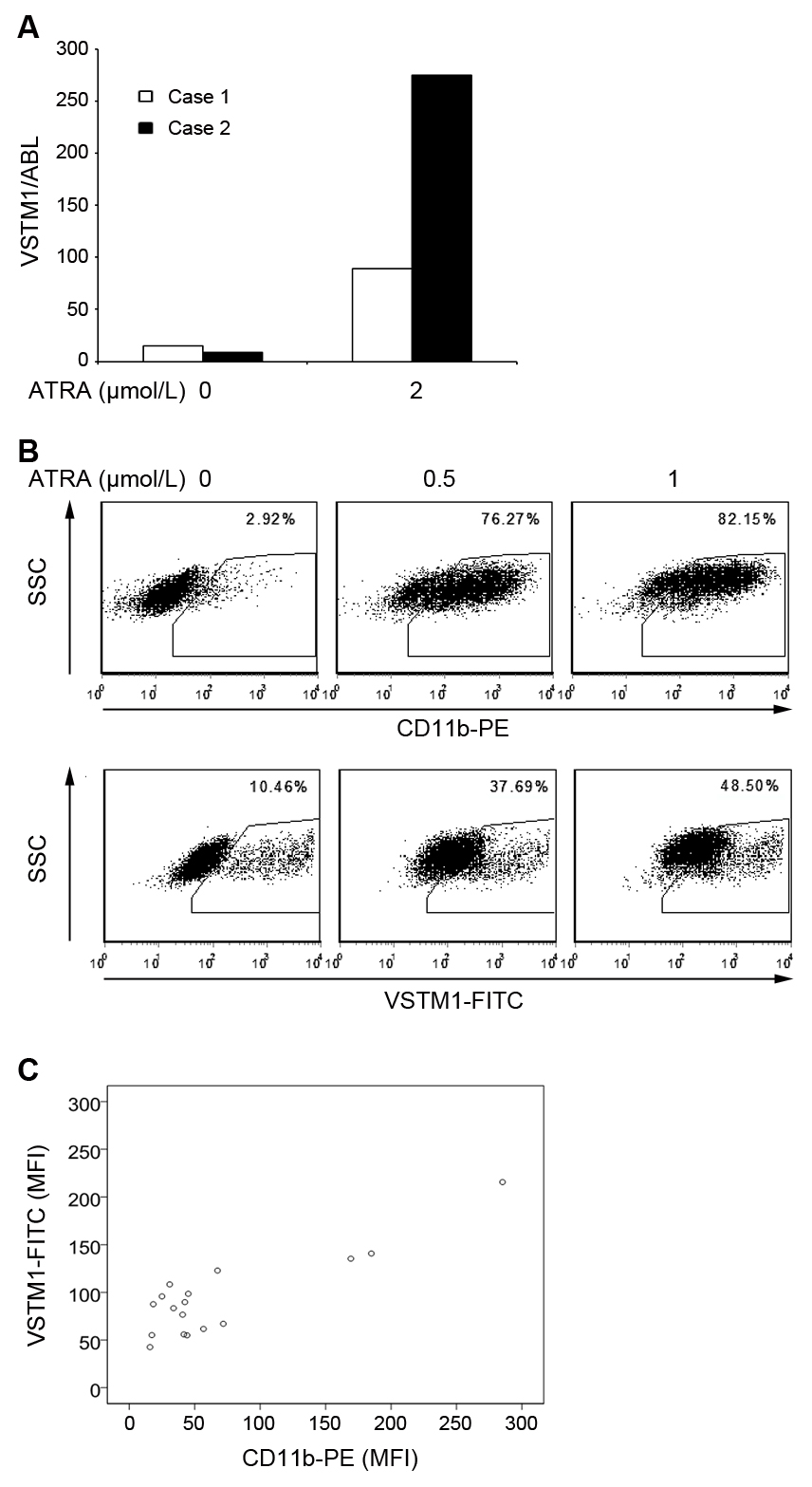

Supplement: Additional file 5: Figure S4. — VSTM1-v1 expression was restored in APL cells by ATRA treatment. (A) qRT-PCR for VSTM1 expression in bone marrow cells from two APL patients that were untreated or treated with ATRA for 7 days in vitro. (B) Flow cytometric analysis of the percentage of VSTM1-v1-positive NB4 cells that were untreated or treated with ATRA for 5 days. CD11b expression was measured as a marker of differentiation. A representative result of three independent experiments is shown. (C) The correlation of the MFI of VSTM1-v1 and CD11b at various time points following treatments with different concentrations of ATRA was evaluated by Pearson’s correlation analysis (n = 17; r = 0.866; P < 0.001). [file 13045_2015_118_MOESM5_ESM.jpg]

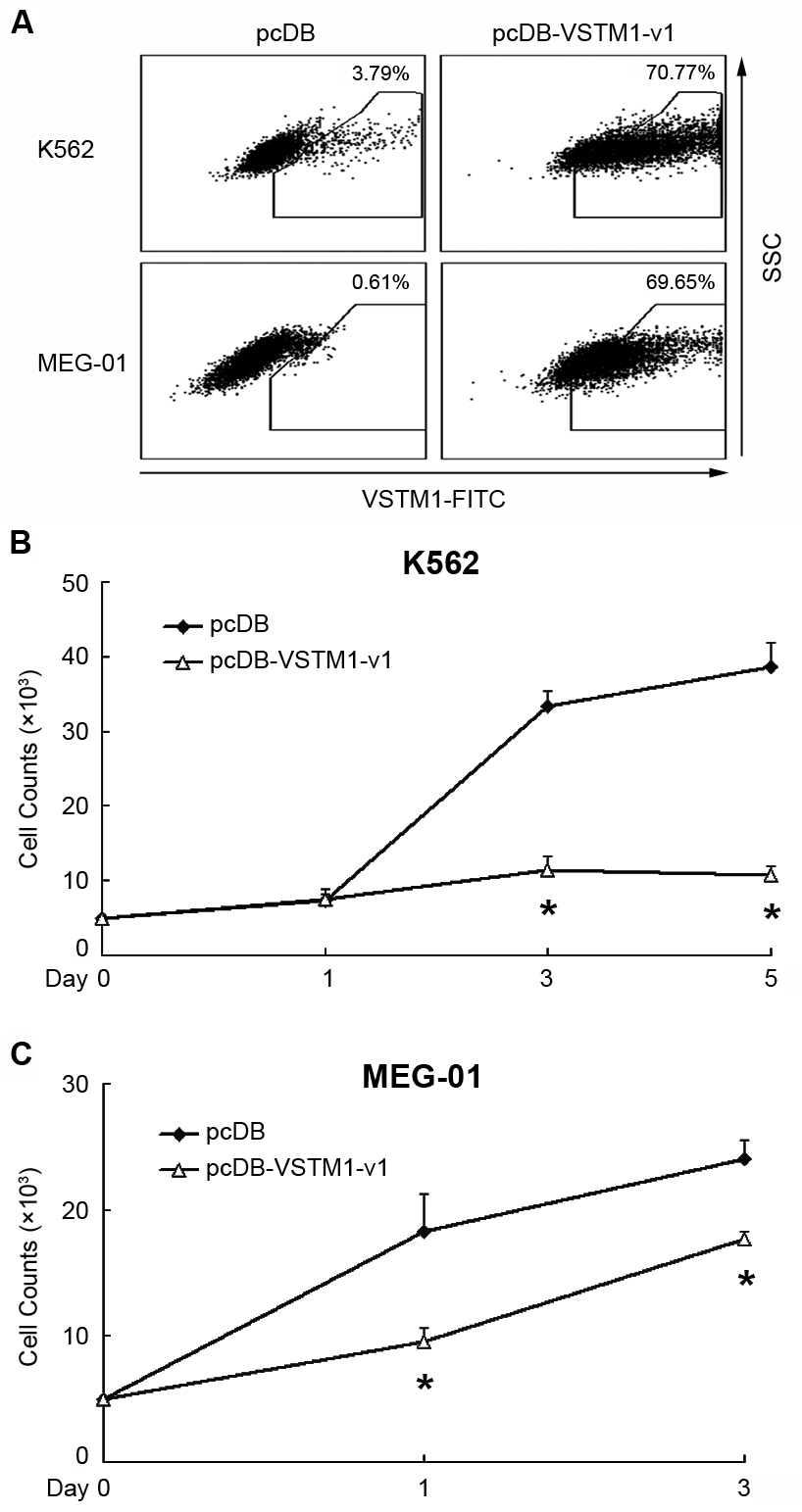

Supplement: Additional file 6: Figure S5. — Overexpression of VSTM1-v1 inhibited K562 and MEG-01 cell growth. (A) Overexpressed VSTM1-v1 was detected on the cell surface by flow cytometry. Cell growth curves of K562 (B) and MEG-01 (C) cells were generated based on viable cell counting assays after VSTM1-v1 transfection. Error bars represent SD; *, P < 0.05, compared to the vector control at each time point. Representative results of at least three independent experiments are shown. [file 13045_2015_118_MOESM6_ESM.jpg]

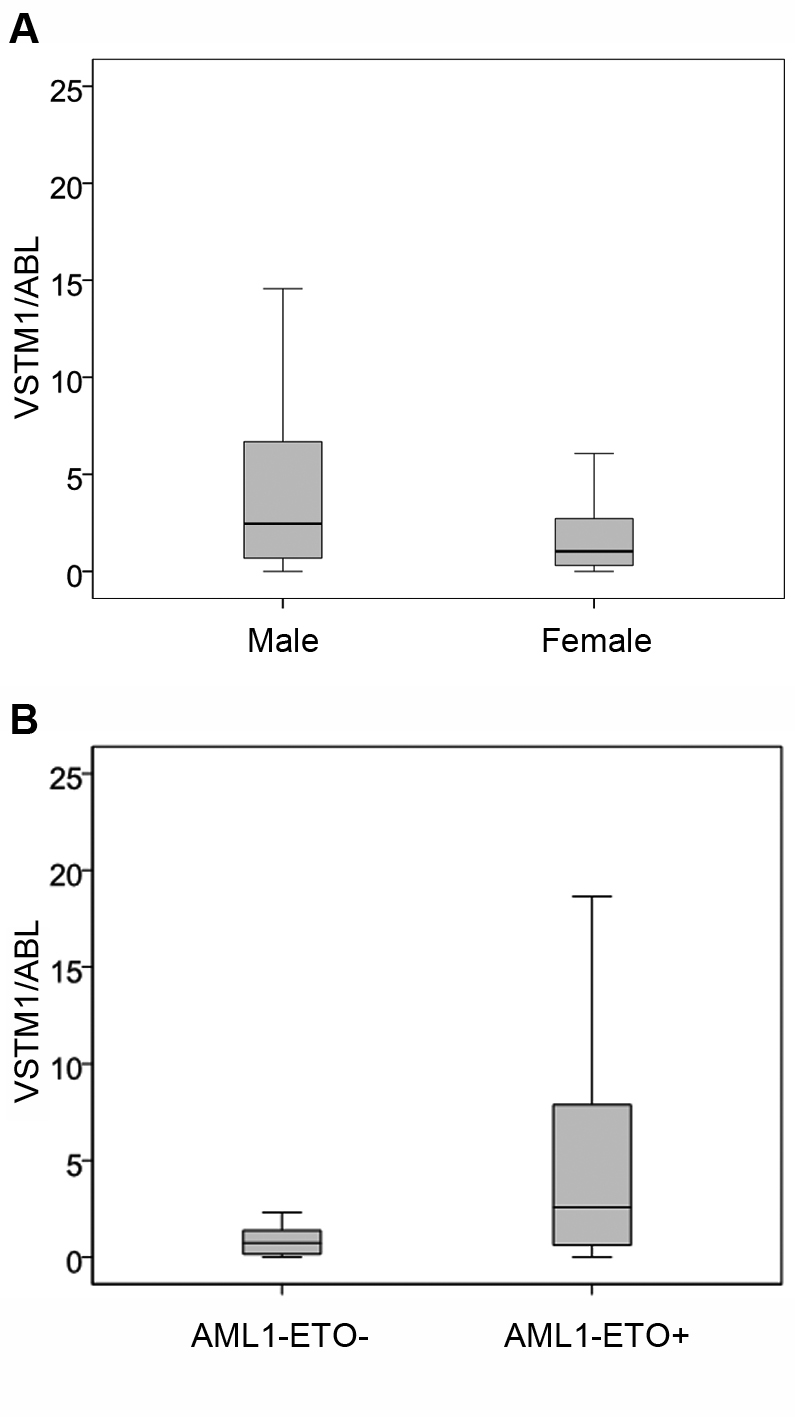

Supplement: Additional file 7: Figure S6. — Correlations between VSTM1 expression in AML patients and gender or the AML1-ETO fusion gene. (A) The expression level of VSTM1 was higher in male than in female patients (median, 2.45 [0.00–171.90] vs. 1.03 [0.00–19.03]; P = 0.010). (B) The expression level of VSTM1 was higher in AML1-ETO-positive (n = 46) than in AML1-ETO-negative (n = 44) patients (median, 2.58 [0.00–44.62] vs. 0.74 [0.00–54.08]; P < 0.001). [file 13045_2015_118_MOESM7_ESM.jpg]
